# Development and efficacy of a novel mRNA cocktail for the delivery of African swine fever virus antigens and induction of immune responses

**DOI:** 10.1128/spectrum.02909-24

**Published:** 2025-04-29

**Authors:** Xing Hu, Cong Liu, Nino Rcheulishvili, Yunzhi Wang, Ting Xiong, Fengfei Xie, Xingyun Wang, Ruiai Chen, Peng George Wang, Yunjiao He

**Affiliations:** 1 Department of Pharmacology, School of Medicine, Southern University of Science and Technology School of Medicine 639321 , Shenzhen, China; 2 Institute of Microbiology, Chinese Academy of Sciences 85387, Beijing, China; 3 Zhaoqing Branch Center of Guangdong Laboratory for Lingnan Modern Agricultural Science and Technology, Zhaoqing, China; 4 Key University Laboratory of Metabolism and Health of Guangdong, Southern University of Science and Technology 255310, Shenzhen, China; Barnard College, Columbia University, New York, New York, USA

**Keywords:** African swine fewer, mRNA vaccine, LNP

## Abstract

African swine fever (ASF) is a highly lethal infectious disease affecting pigs. Although several vaccine formulations have been developed to protect from ASF virus (ASFV) infection, none have yet provided complete protection without side effects or the risk of progressing to chronic infection. mRNA vaccines offer unparalleled advantages in terms of safety and ability to induce immune responses. In this study, we designed six mRNA vaccines encoding key antigenic proteins of ASFV- B602L, CD2V, EP153R, P30, P54, and P72 and combined them into an mRNA cocktail for vaccination in mice and pigs. Our findings suggest that the mRNA cocktail is capable of provoking robust multivalent humoral and cellular immune responses while maintaining a favorable safety profile. Thus, it may serve as a potential approach for controlling ASF transmission, contributing to the ongoing efforts to develop effective and safe ASFV vaccines. The administration of the mRNA cocktail induced both humoral and cellular immune responses in mice and pigs, suggesting a potential for future ASFV vaccine development.

**IMPORTANCE:**

This study explores an mRNA vaccine encoding six critical African swine fever virus (ASFV) antigens (B602L, CD2V, EP153R, P30, P54, P72), demonstrating its ability to induce robust humoral and cellular immune responses in both mice and pigs. This innovative approach serves as a significant advancement in ASFV vaccine development by addressing safety and efficacy concerns. The findings suggest that the mRNA cocktail developed in this study represents a step forward in ASFV vaccine research and development. This strategy holds promise for contributing to ASFV control by offering possibly safer and more effective alternatives to conventional vaccines. This could significantly impact ASF management and prevention strategies globally, ultimately benefiting animal health and reducing economic losses.

## INTRODUCTION

African swine fever (ASF) is a hemorrhagic disease of the panzootic dimension caused by the African swine fever virus (ASFV) and characterized by extremely high infectivity and fatality rates in domestic pigs (1, 2). The clinical manifestations include high fever, anorexia, wheezing, and nasal and ocular discharge, alongside gastrointestinal reactions. These symptoms are often accompanied by general cyanosis. Additionally, in pregnant sows, the infection may lead to spontaneous abortion (1, 3, 4). International collaboration for ASF control involves organizations like the World Organization for Animal Health (WOAH) and the Food and Agriculture Organization to manage and mitigate spread. The WOAH categorizes ASF as a highly severe (List A) disease known for its potential to cause up to 100% fatality rates in pigs (5). In the past 100 years, ASFV has spread from Africa to Europe and Asia, causing huge losses in the local pig farming sector and related industries (1, 6). Particularly in China, the outbreaks of ASF have led to significant economic and gross domestic product losses. As of mid-2019, the ASF outbreak had resulted in the death of 13,355 pigs due to infection. In efforts to control the spread of the virus, a total of 1,204,281 pigs were culled by corresponding authorities. The estimated economic impact of the ASF outbreak amounts to approximately USD 111.2 billion, which represents 0.78% of China’s gross domestic product in 2019 (5).

ASFV has a large size and a complex structure. It is a DNA virus consisting of an envelope, capsid, inner capsid membrane, core shell, and inner core. Its genome spanning about 170–190 kb encodes more than 150 proteins, of which 68 are structural (7). These proteins assemble the virus into icosahedral multilayer structures over 260 nm in diameter (8, 9). A variety of proteins in ASFV are involved in virus infection and immune escape, but the functions of many ASFV proteins and their interactions with host cell membranes are not fully understood (9, 10). For these reasons, developing an ASF vaccine is very challenging. There are two live-attenuated vaccines in the field tests, out of which ASFV-G-ΔI177L is developed by the United States Department of Agriculture in collaboration with National Veterinary Joint Stock Company, which is the first commercial ASF vaccine in the world (11). Another vaccine, ASFV genotype II modified live vaccine (MLV) was developed by the Pirbright Institute. Although in a field study, ASFV-G-ΔI177L-vaccinated Vietnamese breed pigs were partially protected at 2 weeks post-immunization and fully protected 4 weeks after vaccination following the Vietnamese virulent ASFV genotype II strain challenge, there is evidence of virus shedding (12). The MLV vaccine has shown to be promising in protecting from ASFV genotype II ASFV and partially protecting against ASFV genotype I (11). However, the risk of side effects, which are common in the case of live attenuated vaccines, may arise. Premature field testing of the live ASF vaccines may lead to the prolonged forms of the disease characterized by prolonged viral shedding and delayed symptoms, often remaining below the detection limit, worsening the situation (13). Recognizing the shortcomings of traditional vaccines, such as significant side effects and risk of virus virulence reversion, researchers have focused on developing new vaccines against specific targets of ASFV (10). Remarkably, proteins P30, P54, and P72 play a crucial role in various stages of virus attachment and internalization and are capable of inducing the production of neutralizing antibodies. These proteins are recognized for their strong immunogenicity, making them effective antigens (14, 15). B602L protein in the ASFV acts as a molecular chaperone for the P72 protein promoting its correct folding (9). P72, on the contrary, is a major capsid protein that plays a crucial role during the virus assembly process, as it is a key component in the formation of the icosahedral capsid (16). Another protein CD2V can mediate the adhesion of the virus to red blood cells (17), which is related to the immune escape of the virus (18). Additionally, recombinant CD2V proteins expressed with baculovirus showed cross-protection upon challenge (19). Another crucial ASFV antigen EP153R can regulate major histocompatibility complex (MHC) I and inhibit its induced apoptosis during ASFV infection (20). Based on the information above, the selection of B602L, CD2V, EP153R, P30, P54, and P72 antigens seems to be a rational approach for the development of a next-generation effective vaccine. Notably, neutralizing antibodies alone do not appear to be sufficient to protect pigs from challenge death (21). Cellular immunity, particularly CD8+T cell immunity, plays an important role in resisting ASFV infection (22, 23). Thus, these factors need to be addressed when designing a new, effective vaccine.

Various forms of vaccines, such as inactivated, live attenuated, DNA, protein, as well as vector vaccines, have been developed to overcome the ASFV challenge. However, none has successfully contained the spread of ASF without further infection transmission (24, 25). The mRNA vaccine approach known for its efficacy, safety, and cost and time efficiency has garnered significant attention in the field of vaccine development against infectious diseases (26, 27). Indeed, the mRNA vaccine strategy has been successfully used for the prevention of various viral and bacterial infections in preclinical (28–31) and clinical (32–34) studies, as well as commercially available coronavirus disease 2019 vaccines (35). However, as of now, the mRNA vaccine technology has not yet been applied in the development of the ASFV vaccine. Therefore, we have formulated an mRNA cocktail that targets six critical antigens of ASFV: B602L, CD2V, EP153R, P30, P54, and P72. The antigen sequences of the Pig/HLJ/2018 strain were used for the design of the mRNA vaccine. These antigens have been modified by incorporating adjuvants to enhance the efficacy. We finally combined all the mRNAs to obtain the mRNA cocktail, which was administered to the mice and pigs, to evaluate immune responses. The mRNA cocktail induced both humoral and cellular immunities in mice and pigs, marking a step forward in the development of a potential ASFV vaccine.

## MATERIALS AND METHODS

### Cell culture and animal models

HEK293T cells [American Type Culture Collection (CRL-3216)] were cultured in 10% FBS Dulbecco's Modified Eagle Medium (Thermo Scientific, USA) at 37℃ in a 5% CO_2_ incubator. Female BALB/c mice (6 to 8 weeks old, specific pathogen-free) were obtained from Guangdong Yaokang Biotechnology Co., Ltd., Foshan. Pigs were provided by Guangdong Wens Dahuanong Biotechnology Co., Ltd. The positive sera were purchased from the China Veterinary Drug Supervision Institute.

### Peptides and proteins

Peptides corresponding to the predicted epitopes were chemically synthesized (Sangon, Shanghai, China) (see File S1 for all synthesized peptides). These synthetic peptides were dissolved in sterile phosphate-buffered saline (PBS) to a concentration of 2 mg/mL to create peptide stocks. The stocks were aliquoted and subsequently stored at −20℃ until use.

### Recombinant vectors

The pVAX1(+) plasmid vector was designed and synthesized for the RNA *in vitro* synthesis (GENEWIZ, Suzhou, China). As a signal peptide, a sequence of a tissue plasminogen activator (tPA) was incorporated upstream of the open reading frames (ORFs) to enhance the expression of ASFV tandem and its secretion from the cell. The following 5′ UTR sequence was used: GAGAATAAACTAGTATTCTTCTGGTCCCCACAGACTCAGAGAGAACCC. As for the 3′ UTR sequence, it was obtained from a patent (US (PCT/US2020/022710) of Moderna, Inc.

### Expression and purification of target proteins

Six protein-encoding genes were cloned into the pET28a expression vector (Invitrogen, US) with a C-terminal 6× His tag and transformed into BL21(DE3) pLysS competent cells. A single colony carrying the target protein particles expressed recombinant proteins carrying six consecutive His residues under IPTG induction at 37℃. The cells collected by centrifugation were crushed using a high-pressure homogenizer, and the supernatant obtained after high-speed centrifugation was first purified by NTA-Ni affinity chromatography, then purified by size-exclusion chromatography.

### mRNA synthesis

The antigen sequences obtained from the Pig/HLJ/2018 strain are given in File S2. mRNA molecules were synthesized *in vitro* via T7 polymerase-mediated RNA transcription. The ORFs were codon-optimized along with flanking 5′ and 3′ UTRs, and a poly-A tail was obtained from GENEWIZ. *In vitro* transcription utilized linearized plasmid DNA as a template. Modified nucleoside-containing mRNA was produced using N1-methylpseudouridine (m1Ψ) triphosphate in place of uridine triphosphate (UTP). The 5′ end of the mRNA was capped with Cap1 (Glycogene, Wuhan, China). The mRNA was purified using a Monarch RNA Cleanup Kit (New England Biolabs, Ipswich, MA, USA). *In vitro* produced mRNA concentration and purity were detected by Nanodrop 2000c (Thermo Fisher Scientific).

### Preparation and quality control of mRNA-LNP

Lipid nanoparticles (LNPs) were produced by combining one volume of lipid mixture consisting of an ionizable cationic lipid, phosphatidylcholine, cholesterol, and polyethylene glycol lipid in a molar ratio of 50:10:38.5:1.5. mRNA was dissolved in citrate buffer (pH = 4.0). The lipids and mRNA were mixed using a T-mixer (Inano E, Micro & Nano Technology, Inc., Shanghai, China) at a ratio of 1:3. Subsequently, the formulations were dialyzed against PBS (pH = 7.4) using pre-sterilized Amicon Ultra-15 centrifugal filters (Millipore, Burlington, NJ, USA) for ethanol removal for 30 min, three times. The final mRNA preparation was sterilized through a 0.22 µm filter and stored at 4℃ until its use.

Quality control assessments for mRNA-LNP encompassed encapsulation efficiency, zeta potential, average particle size, and particle size distribution. The RiboGreen RNA Detection Kit (Thermo) was used to determine the mRNA-LNP encapsulation efficiency according to the manufacturer’s instructions. The Malvern Zetasizer was used to determine the mRNA-LNP particle size and particle size distribution. Zeta potential was evaluated using a Zetasizer Nano ZS (Malvern Panalytical) System.

### Transfection of mRNA into HEK293T cells

Transfection of HEK293T was done using Lipofectamine 2000 (Thermo Fisher Scientific) following the manufacturer’s instructions. Before transfection, the culture medium was replaced with Opti-MEM (Gibco). Next, 2.5 µg of mRNA-Tandem was diluted in 250 µL Opti-MEM (Gibco) and mixed with Lipofectamine 2000 (5 μL). After a 15 min incubation at room temperature, the mixtures were introduced into the cell culture media. Subsequently, the lysates and supernatants were harvested for protein analyses.

### Western blot

Western blot analysis was conducted on whole-cell lysates and supernatants from cells transfected with mRNA (B602L, CD2V, EP153R, P30, P54, and P72). A mixture of the samples with 4× loading buffer was prepared. The separation was done in 4–20% SurePAGE, and then transferring to polyvinylidene difluoride membrane using the eBlotL1 Efficient Wet Blotting Transfer System (GenScript, Nanjing, China) was conducted. The blocking of the membrane was achieved using 5% bovine serum albumin (BSA) in 1× phosphate-buffered saline 0.1% Tween (PBST) buffer. ASFV tandems were detected via 1:2,000 anti-GM CSF antibody (SinoBiological, Beijing, China) by incubation for 2 h. Following this, the membranes underwent three washes with PBST and were incubated with goat anti-rabbit IgG-HRP at a 1:2,000 dilution (Abcam, Cambridgeshire, UK) for 1 h. Visualization was done with the Tanon 5200 Chemiluminescent Image System.

### Mouse experiments

To assess the immunological effects of mRNA-Tandem *in vivo*, female BALB/c mice (age: 6–8 weeks) received intramuscular immunizations with mRNA-Tandem-LNPs (dosage: 30 µg) or empty LNP (*n* = 6) as a control group. The immunization regimen included a primary dose, followed by a booster. Serum samples were subsequently collected and kept at −20℃. The spleen lymphocytes were harvested on day 42 to evaluate the cellular immune responses via ELISpot and flow cytometry analysis.

### Pig experiments

A total of 12 female pigs (8 weeks old) were included in this study. The non-vaccinated group served as the control. Pigs received intramuscular immunizations with mRNA-Tandem-LNPs (dosage: 180 µg) or LNPs alone (*n* = 6) as a control. The immunization protocol included two initial doses, followed by a booster. To assess humoral and cellular immune responses, serum samples were collected and analyzed using enzyme-linked immunosorbent assay (ELISA), while peripheral blood mononuclear cells (PBMCs) were obtained for the ELISpot assay. For ELISA, the blood was allowed to clot, and serum was separated and used for the analysis.

### ELISA

IgG expression assessment was conducted by ELISA. The 96-well ELISA plates (Corning, New York, NY, USA) were coated with 2 µg/mL of each protein (B602L, CD2V, EP153R, P30, P54, and P72) and incubated overnight at 4℃. After overnight coating, plates were blocked with 5% BSA in PBST at 37℃ for 1 h to prevent non-specific binding. Following two PBST washes, diluted mouse sera were added to the plates and incubated for 2 h at room temperature, followed by three washes with PBST. Next, the plates were incubated with an HRP-conjugated anti-mouse IgG antibody at a dilution of 1:10,000 (Abcam, Cambridge, UK), followed by the addition of TMB substrate (Beyotime) for color development. The absorbance at 450 nm was quantified using a Synergy HTX microplate reader (BioTeK, Winooski, VT, USA).

### ELISpot

Mice splenocytes were prepared by forcing shredded spleen through a cell strainer (Falcon, NY, USA). After lysing red blood cells with the ACK lysing buffer (Thermo), the splenocytes were washed extensively with PBS, whose viability was >90% after treatment. The T cell immune response to epitopes was performed by using the Mouse IFN-γ Precoated ELISpot Kit (Dakewe, Shenzhen, China) following the manufacturer’s instructions. Cells were plated in duplicate at 4 × 10^5^ per well and incubated for 18 h in a final volume of 100 µL RPMI 160. The predicted T cell epitope peptides in vaccines were used as stimulators (17 in total) at a final concentration of 10 µg/mL. Cells were lysed by incubating for 10 min in cold water and washed with washing buffer. Biotinylated anti-mouse IFN-γ antibodies subsequently detected with streptavidin–HRP conjugates were used for spot visualization, which were quantified using the ELISpot Reader System, AID. Spot counts were normalized to per million cells, and the mean from duplicate wells was plotted. The evaluation of the effect of each T cell epitope was also carried out by ELISpot. In case of pigs, PBMCs were used instead of splenocytes in the ELISpot assay. PBMCs from the peripheral blood of pigs were isolated via density gradient centrifugation. The isolated PBMCs were then washed and resuspended in RPMI 1640 medium supplemented with 10% fetal bovine serum. The porcine PBMCs were plated in duplicate at a density of 4 × 10⁵ cells/well and incubated with the predicted T cell epitope peptides (final concentration of 10 µg/mL) in a final volume of 100 µL RPMI 1640 for 18 h. After incubation, the cells were lysed, and the subsequent steps of biotinylated anti-IFN-γ antibody incubation, streptavidin–HRP conjugate detection, and spot visualization were carried out following the same principles as in the mouse experiment.

### Flow cytometry

Antigen-specific T cell responses were analyzed by flow cytometry using prepared splenocytes on a multicolor flow cytometer (BD Biosciences). Cells were counted and added in 2 million per well (100 µL). Each sample was stimulated with the T cell epitope mix as previously described for 8 h at 37℃. Brefeldin A (Thermo Scientific) was then added into cells for 4 h of incubation. Cells, after stimulation, were rinsed in PBS containing 0.5% BSA. TruStain FcXTM antibody (BioLegend) was used to reduce non-specific staining of cell surfaces. T cell surface markers were labeled using APC/Fire 750 anti-mouse CD3 antibody (BioLegend, San Diego, CA, USA), FITC anti-mouse CD4 antibody, and Brilliant Violet 510 anti-mouse CD8a antibody. Following this, cells were fixed using the Fixation/Permeabilization Solution Kit (BD Biosciences) and individually stained with PE anti-mouse IFN-γ, PE anti-mouse IL-2, and PE anti-mouse IL-4 antibodies. Data were analyzed using FlowJo (V10.0.7). The gating strategy for the flow cytometry of CD4 and CD8 T cells is given in File S3. The antibody clone information for each used antibody is given in File S4.

### Statistical analysis

Data analysis was conducted using GraphPad Prism version 8.0. Across all experiments, the data are displayed as mean ± standard errors. Given the small number of samples and groups available for testing, all statistical analyses were performed by *t*-tests. *<*I*>*P* < 0.05; **<*I>P* < 0.01; ***<*I>P* < 0.001.

## RESULTS

### Design and characterization of the mRNA cocktail

**Fig 1:**
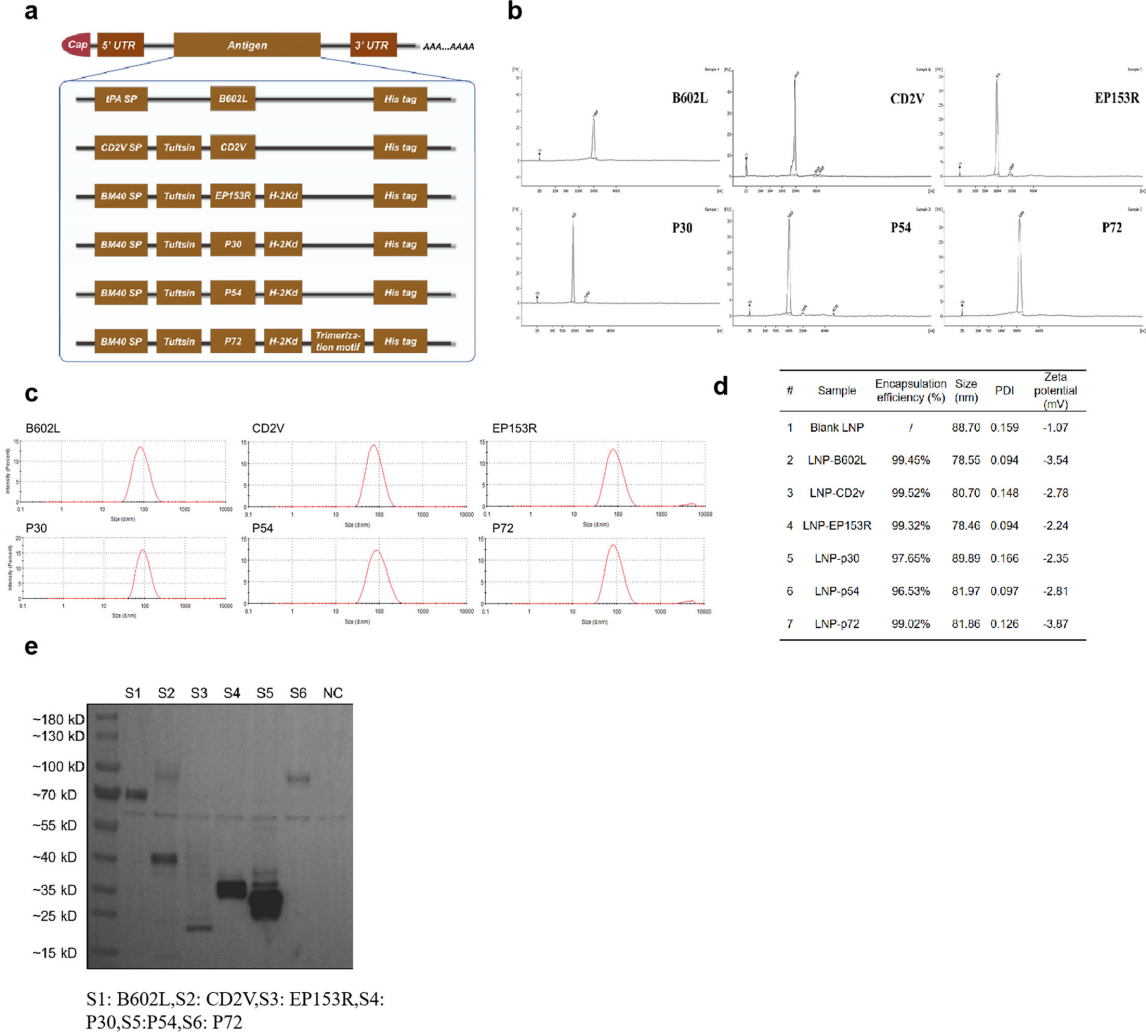
Design and characterization of the mRNA cocktail. (a) Schematic diagram of the mRNA vaccine structures and encoded antigen composition. (b) Six mRNA analysis by capillary electrophoresis. (c) Particle size of six kinds of mRNA-LNP. (d) Characterization of six mRNA-LNPs. (e) Expression results of six mRNAs in HEK293T cells.

mRNA vaccines usually include Cap, 3′ and 5′ UTRs, ORF, and Poly-A tail. In our mRNA vaccine, we used a unified Cap 1 (CleanCap Reagent AG), along with 3′ and 5′ UTRs, and a 100 nt Poly-A tail. As for the ORF, we incorporated six ASFV antigens (B602L, CD2V, EP153R, P30, P54, and P72), enhancing them with a series of elements, such as secretion signal peptide (36), to facilitate protein secretion, molecular adjuvants like tuftsin (Thr-Lys-Pro-Arg, a natural immunomodulating peptide) (37), H-2Kd (TYQRTRALV, a Kd-restricted epitope from influenza nucleoprotein) (38) in order to enhance cellular immune responses, and His tag to facilitate protein detection. For the mRNA of antigen P72, a trimerization motif from T4 bacteriophage fibrin (GYIPEAPRDGQAYVRKDGEWVLLSTFL) was incorporated with the expectation that it would induce the formation of the P72 trimeric structure (Fig. 1a) (39). The constructed sequence was introduced into the pVAX1 vector. mRNA vaccines were synthesized via T7 polymerase-mediated *in vitro* transcription with N1-methyl-pseudouridine nucleoside substituting uridine (U). The synthesized mRNA molecules were shown to be of high integrity as determined by capillary electrophoresis (Fig. 1b). mRNA was encapsulated with LNP using a microfluidic system for delivery *in vivo*, and the average size of resulting particles detected by dynamic light scattering was about 80  nm (Fig. 1c), with the polydispersity index lower than 0.2 (Fig. 1d). The encapsulation efficiencies of mRNA-LNP were higher than 95% as detected by the Quant-iT RiboGreen RNA Kit. Transfection of HEK293T cells with mRNA achieved the expression of target proteins as detected by the anti-His antibody (Fig. 1e).

### mRNA cocktail vaccine induced immune responses in mice

**Fig 2:**
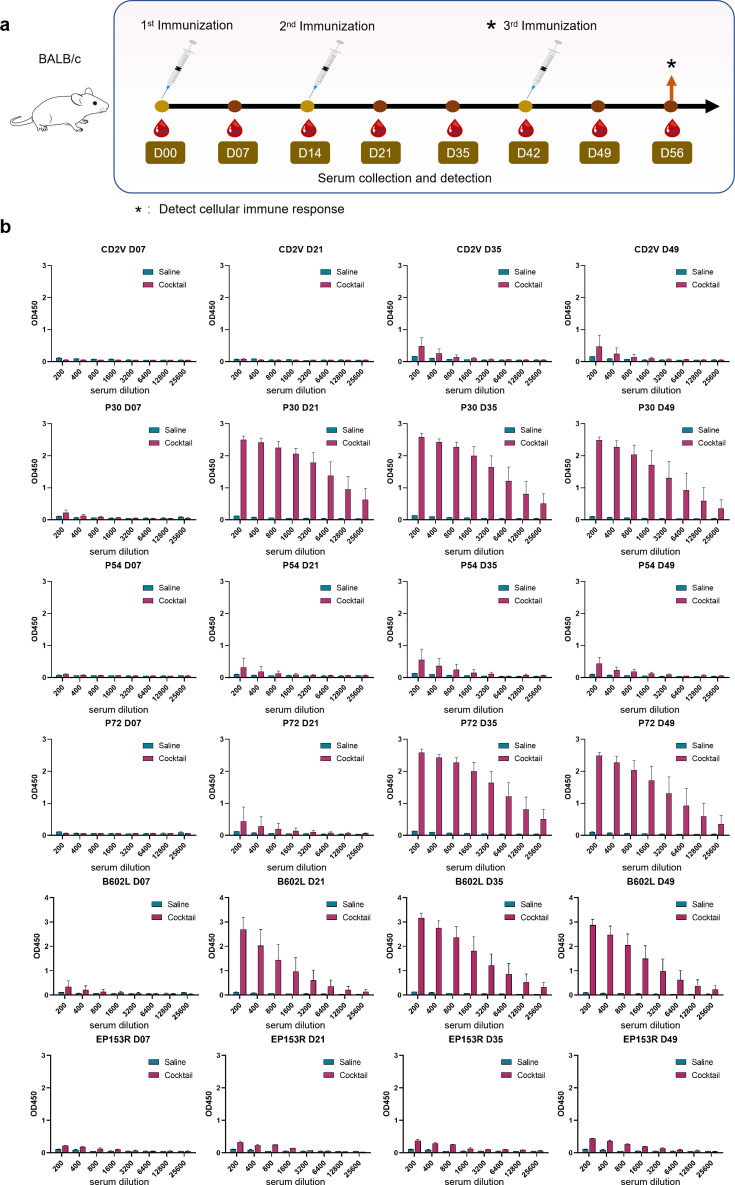
Mice immunization scheme and vaccine-induced humoral immune response in mice. (a) Strategy of mRNA vaccine administration in mice and scheme of detecting humoral and cellular immune responses. (b) Changes in antibody levels to specific antigens in mice that received multiple immunizations.

We performed a total of three immunizations on experimental mice. Blood was collected at weekly intervals for antibody detection. The indicators for evaluating the humoral immune response used several target proteins known to produce antigen-specific antibodies in current vaccines (Fig. 2a). Vaccinated mice were sacrificed 28 days post-second immunization and 14 days following the third immunization, and their spleen cells were used for ELISpot assay and flow cytometry (Fig. 2a). Mouse spleen cells were stimulated overnight with the selected T cell epitope in the target protein in cocktail to assess the cellular immune response and corresponding cytokine secretion.

The vaccinated mice began to produce large amounts of anti-P30 antibodies 7 days after the first immunization, peaked at 7 days after the second vaccination, and remained stable for 4 weeks thereafter. Antibody levels of the mRNA cocktail vaccine against other antigens, such as P54 and P72, peaked 21 days post-second immunization and eventually produced high levels of antibodies against antigen P72. However, the response of mouse sera to the antigens P54 and CD2V remained weak, and the corresponding antibody levels did not change much 7 days after the third booster immunization (Fig. 2b).

**Fig 3:**
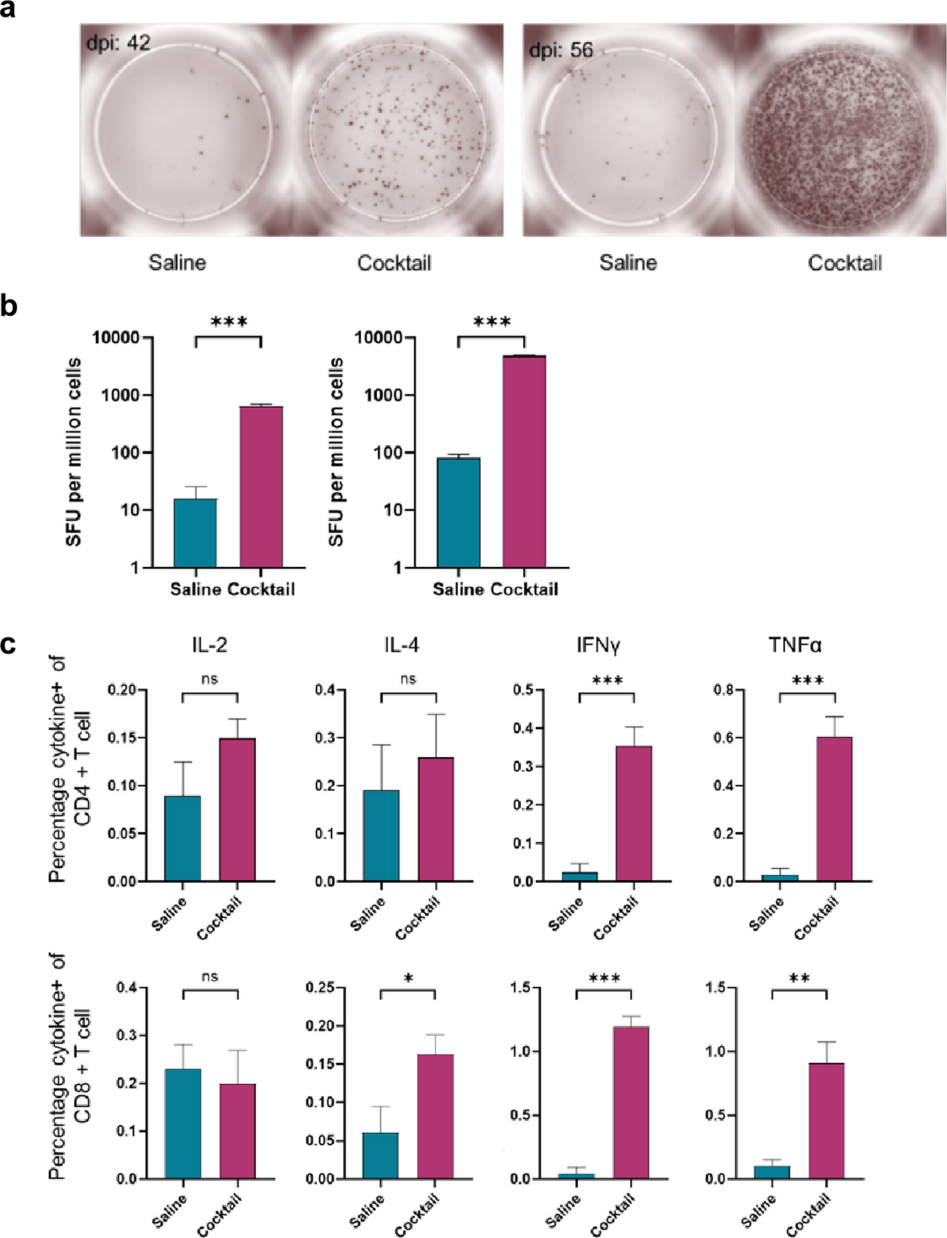
Vaccine-induced cellular immune response in mice. (a) Comparison of spot counts between the control and vaccine groups after the second and third immunizations. (b) Statistical graph of spot counts in the control and vaccine groups after the second and third immunizations. (c) Vaccine-induced cytokine polarization.

Previous studies have shown that virus-specific T-cell immune responses are crucial for controlling ASFV infection. The ELISpot assay is used to assess whether the vaccine induces cellular immune responses by detecting the ability of spleen cells to secrete interferon-gamma (IFN-γ). Accordingly, 28 days after the second immunization, the spleen cells of the mice in the vaccinated group produced an average of 655 spots, and 14 days after the third immunization, the spleen cells of the mice in the vaccine group produced an average of 4,870 spots. The level of IFN-γ secretion in the vaccine group was much higher compared with that in the control group (Fig. 3a and b). The results showed that mice immunized with the vaccine developed a memory for the antigen. Cellular immune responses can be induced by vaccines and can be enhanced by booster immunizations.

In order to accurately assess specific T cell function and response, we detected the ratio of CD4+ and CD8+ cells in the spleen cells of mice by flow cytometry, while intracellular cytokine staining for IL-2, IL-4, IFN-γ, as well as TNF-α, detected cytokines secreted in response to epitope stimulation. From the staining results of the mouse spleen, we found that compared with those produced in saline-treated mice, the intracellular production of IL-4, IFN-γ, and TNF-α was much higher in both CD4(+) and CD8(+) splenocytes of inoculated mice, while the intracellular production of IL-2 was higher in CD4(+) T cells but lower in CD8(+) T cells of immunized mice after the stimulation of corresponding peptides (Fig. 3c). T cell responses to each individual antigen in mice are given in File S5.

### The mRNA cocktail vaccine induced physiological and immune responses in pigs

**Fig 4:**
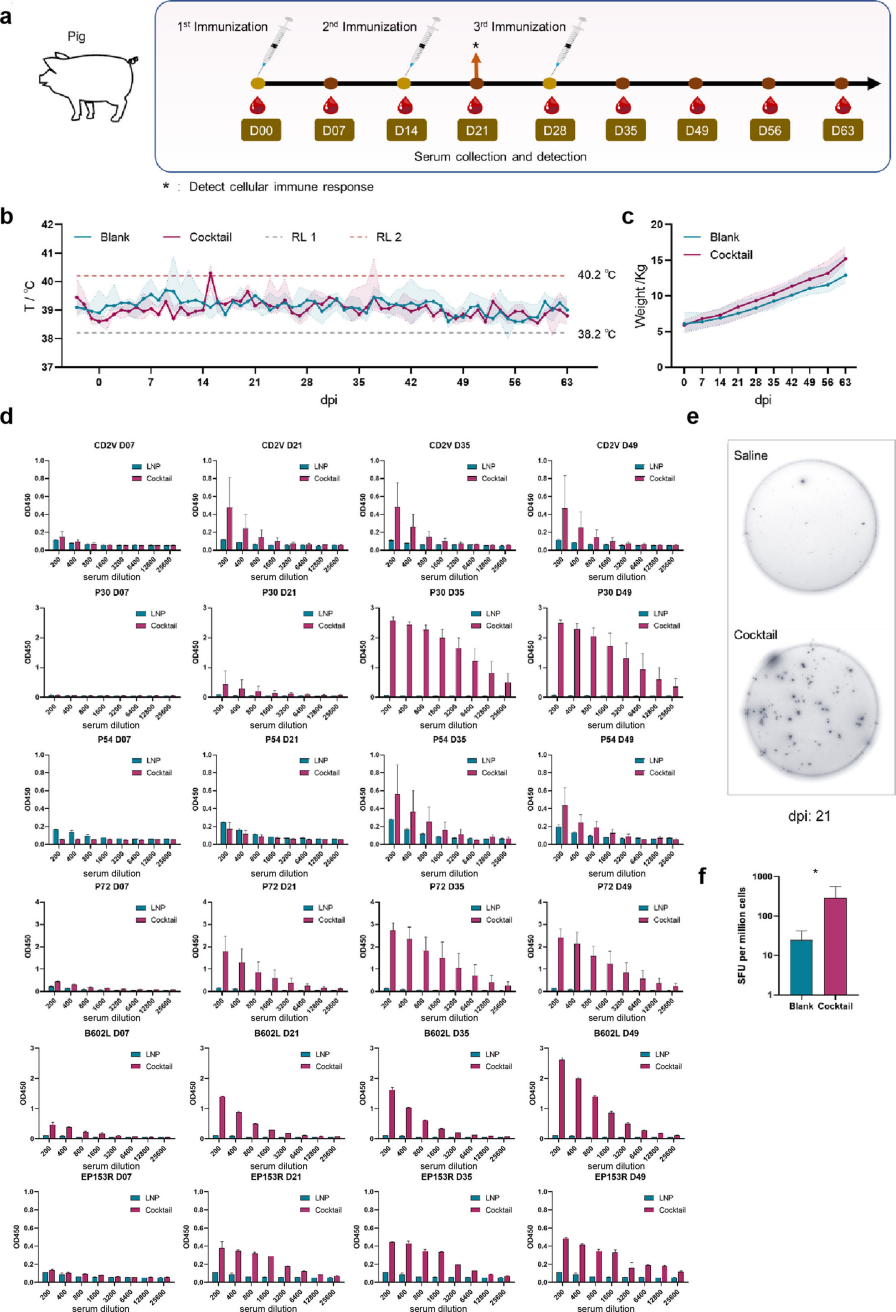
Vaccine-induced physiological and immune responses in pigs. (a) Strategy for immunizing pigs with mRNA vaccines and plan for assessing humoral and cellular immune responses. (b) Trends of daily body temperature changes in the control and vaccinated groups. (c) Weekly body weight changes in the control and vaccinated groups. (d) Changes in antibody levels to specific antigens in pigs that received multiple immunizations. (e) Comparison chart of spot counts between the control and vaccinated groups. (f) Statistical chart of spot counts in control and vaccinated groups. dpi, days post infection.

We also tested the safety and efficacy of the vaccine in pigs. A total of three immunizations were carried out on the pigs with an interval of 14 days between immunizations. The body temperature of each pig was recorded daily, and the peripheral blood was collected every 7 days. Additionally, the pigs were weighed at each of these intervals. The ability of the vaccine to induce humoral and cellular immune responses in pigs was tested by ELISA and ELISpot methods (Fig. 4a).

None of the four immunized pigs succumbed during the vaccination period. The body temperature of experimental pigs fluctuated during the immunization period, and the overall body temperature was within the normal temperature range of 38.2–40.2°C (Fig. 4b). The weight of all pigs increased steadily during the immunization period, and there was no significant difference in the weight of the pigs in the vaccine and control groups, suggesting the vaccine did not affect the weight of pigs (Fig. 4c).

We also evaluated the vaccine-induced humoral and cellular immune responses in pigs. The vaccine induced slightly different timings and levels of antibody production against different antigens in both pigs and mice. Antibody levels against P30 did not increase until 7 days post-second immunization and were at higher levels thereafter. Antibody levels against P72 rose steadily until they peaked and stabilized 7 days after the third immunization. Seven days post-second immunization, the immunized group began to produce antibodies against CD2V and P54, but the overall antibody level was only slightly higher than that of the control group (Fig. 4d). Notably, we also found the ability of the vaccine to induce a cellular immune response in pigs. We detected PBMCs from pigs 7 days after the second immunization, stimulated with selected epitope peptides, and the average level of IFN-γ secreted by the vaccine group was about six times that of the control group (Fig. 3c). IFN-γ secretion induced by stimulation of cells with the predicted T cell epitope peptides from each antigen was also assessed via ELISpot assay. Spot counts of IFN-γ-producing T cells detected by ELISpot are given in File S6.

Taken together, these findings suggest that this mRNA–vaccine combination can induce a multivalent humoral immune response and a robust T cell response, offering a promising translatable vaccine candidate for ASFV infection control.

## DISCUSSION

ASFV is highly contagious and lethal to pigs, with a significant impact on the global swine industry due to high mortality rates and economic losses. Currently, there is no effective commercial vaccine available, making the control and eradication of ASFV challenging, especially in countries with large swine populations (11). Given the complexity of ASFV infection and the need for broad and effective immune responses, current vaccine development strategies typically involve selecting multiple target antigens for immunization. It is important to emphasize that cellular immunity plays a crucial role in combating ASFV infection. mRNA vaccines, particularly when formulated with LNP, show promise in eliciting cellular immunity. Additionally, the ORFs of these vaccines offer flexibility for antigen modification and adaptation (26). Indeed, mRNA-based vaccines have ushered in a transformative era in vaccinology characterized by favorable safety, strong immunogenicity, cost-effective production, and rapid development, among other benefits (35). Therefore, mRNA vaccines are particularly suitable for the research and development of vaccines against ASFV infection based on target antigens.

In this study, we selected and combined six target antigens of ASFV (B602L, CD2V, EP153R, P30, P54, and P72), incorporated elements, such as adjuvants, and finally formulated the mRNA cocktail preparation. The B602L protein of ASFV is a promising candidate for inclusion in an mRNA vaccine construct due to its enhanced immunogenicity when fused with an Fc fragment. Yang et al. demonstrated that the B602L-Fc fusion protein can bind and interact with the FcRI receptor on antigen-presenting cells. This interaction significantly enhances the expression of proteins critical for antigen presentation and various cytokines at the mRNA level in porcine alveolar macrophages. This fusion protein also notably promoted a Th1-biased cellular immune response and humoral immune response in mice, suggesting its potential as an effective component in ASFV subunit vaccines (40). The CD2v, ASFV outer envelope protein is an integral component in the development of an effective vaccine. Liu et al. evaluated the safety and immunogenicity of replication-incompetent type-2 adenoviruses carrying ASFV antigens, including CD2v, and showed that a vaccine cocktail containing these antigens elicited robust systemic and mucosal immune responses in mice and swine. This suggests that CD2v is a key antigen for inducing immunity against ASFV, making it a strong candidate for inclusion in mRNA vaccine constructs (41). The EP153R protein, which is a part of the viral capsid, is also an important target for vaccine development due to its role in the pathogenesis of the virus and immune response (11). The p30, a capsid protein located in the inner envelope of ASFV [5], is a crucial immunogenic antigen being considered for inclusion in vaccine constructs due to its role in protective immune responses, making it a valuable target in ongoing ASF vaccine research (11). The p54 protein, a viral inner envelope component, is considered a reasonable candidate for inclusion in mRNA vaccine constructs due to its ability to elicit robust humoral and cellular immune responses. Studies involving adenovirus vector vaccines carrying the p54 protein have demonstrated its potential to stimulate a robust immune response (42, 43). The p72, a major capsid protein located in the outer capsid layer of ASFV, is a key antigen and a suitable candidate for inclusion in mRNA vaccine constructs. Its high immunogenicity and reactivity, along with the identification of linear B cell epitopes within p72, indicate its dominant role and potential in ASF vaccine development. The conservation of these epitopes across various ASFV genotypes further highlights their importance in the development of ASF vaccines (44).

Besides antigens, additional components were incorporated in the mRNA construct to enhance the outcome of the vaccine. The signal peptide is an important part in mRNA vaccines ensuring efficient protein secretion from the cell (36). The tPA signal sequence plays a major role in enhancing the expression of the vaccine antigen by directing the antigen protein to the secretory pathway of the cell. This leads to the efficient secretion of the antigen into the extracellular environment, where it can be better recognized by the immune system (45). Another incorporated sequence, tuftsin, serves as an adjuvant by enhancing immune response. As a natural immunomodulatory peptide, tuftsin stimulates immune cells, such as macrophages, resulting in improved antigen presentation (37). Another molecular adjuvant MHC I protein H-2Kd was used due to its implication in antigen presentation to T cells. We examined the potential of the designed mRNA cocktail vaccine encoding the abovementioned six ASFV antigens by testing its ability to induce immune responses against ASFV infection. During the immunization, antibody production against a variety of antigens was observed both in mice and pigs. The mRNA cocktail vaccine could trigger a strong humoral immune response, but the level of antibodies against each antigen varied. This is not only related to the detection of antigens but also to the expression, translation level, and stability of various mRNAs that were included in the vaccine cocktail. Studies have shown that the stability of mRNA vaccines is a critical factor that can impact their effectiveness. The inherent properties of mRNA vaccines, their interaction with lipid nanoparticles, and factors, such as mRNA structure and manufacturing processes, are pivotal in determining vaccine stability and, consequently, the capacity to elicit immune response (46). Besides, different antigens in a vaccine can induce varied immune responses. Indeed, Chivukula et al. performed research on the development of multivalent mRNA vaccine candidates for influenza and demonstrated that distinct antigens can trigger different immune responses. This variation in response can be observed in the levels of antibodies produced against different antigens present in the vaccine formulation (47). Hence, both the efficiency of the mRNA delivery and the selection of optimal formulation parameters are crucial in achieving effective immune responses. The understanding of the immunogenicity of different antigens within mRNA vaccine formulations is very important, as this can significantly impact the overall vaccine efficacy.

Additionally, a significant release of IFN-γ was observed from the immune cells of both mice and pigs. In the spleen cells of mice, the vaccine group exhibited considerably higher levels of TNF-α secretion compared to the control group. IFN-γ and TNF-α are important pro-inflammatory cytokines in immunity to ASF infection, which can regulate, activate, and enhance cellular immune response (48). The vaccine mediated a strong secretion of IFN-γ and other cytokines, demonstrating its ability to induce cellular immune responses against ASFV proteins. The secretion of IL-2 and IL-4 was also observed, although not at significantly higher levels compared to the control group. IL-2 is primarily involved in the growth, proliferation, and differentiation of T cells (49), while IL-4, also called "prototypic immunoregulatory cytokine,” regulates antibody production, hematopoiesis, and the development of effector T-cell responses (50). Our results show that the humoral and cellular immune responses induced by the vaccine have the potential to synergistically contribute to the resistance to ASFV infection. It is worth noting that the pigs immunized with the vaccine did not die or suffer from serious adverse symptoms during the experiment, and their body temperature rose only for a short time. This suggests that our vaccine combination and dose setting are relatively safe.

Despite the encouraging results, it is important to address the limitations of the study, which lie in three main areas. One significant limitation of our study is the uncertainty regarding the functionality of the H-2Kd-restricted T cell epitope in pigs. Currently, there is a lack of research specifically focused on the interaction between this epitope and the swine leukocyte antigen (SLA) in the context of ASFV vaccines. As a result, there is a lack of direct evidence to confirm its effective presentation by SLA with high affinity. This knowledge gap may potentially impact the optimization of the cellular immune response induced by our mRNA cocktail in pigs. Future studies should address this issue to enhance the understanding and effectiveness of ASFV vaccines targeting the porcine population. In terms of design, another limitation is that the initial optimization of antigen expression and the immunization dose were not fully addressed. The mRNA vaccine essentially needs to be translated into a protein to be effective. The effective expression and induction of an antigenic immune response critically depend on the reasonable design of the mRNA–antigen and the appropriate determination of the dosage (26). Ultimately, while the current study shows promising immunogenicity and safety in both mice and pigs, the absence of a challenge experiment due to the lack of an ABSL-3 facility limits the ability to fully evaluate the protective efficacy of the vaccine (51). Furthermore, previous studies reported immune responses to the various ASFV antigens formulated with different vaccine platforms without conferring full protection against ASFV infection (52, 53). This highlights the need for further research to determine whether the immune responses induced by our mRNA cocktail will be translated into effective protection. Future studies, including challenge experiments in vaccinated animals, will be crucial to validate these findings and assess the true potential of this approach for practical application in controlling ASFV.

In conclusion, the current study demonstrates promising preliminary results in an attempt to develop an effective vaccine for ASF. The mRNA cocktail vaccine tested in both mice and pigs elicited robust humoral and cellular immune responses while maintaining a favorable safety profile. These findings highlight the potential of this mRNA cocktail as a viable and safe option for controlling ASF transmission. Conducting further research addressing the limitations of the study is essential to fulfill the urgent need for effective and fully protective ASFV vaccine.

## Data Availability

Data used in this article may be shared with other investigators upon written request to the corresponding author.

## SUPPLEMENTAL MATERIAL

Supplemental material spectrum.02909-24-s0001.pdf Two supplemental tables; supplemental data; three supplemental figures.

ASM does not own the copyrights to Supplemental Material that may be linked to, or accessed through, an article. The authors have granted ASM a non-exclusive, world-wide license to publish the Supplemental Material files. Please contact the corresponding author directly for reuse.
