# Supplementary material for: Development and efficacy of a novel mRNA cocktail for the delivery of African swine fever virus antigens and induction of immune responses
Source: Microbiol Spectr. 2025 Apr 29;13(6):e02909-24. doi: 10.1128/spectrum.02909-24 (PMC12131786; doi:10.1128/spectrum.02909-24)

Supplementary materials

Supplementary file S1. Synthesized peptides corresponding to the predicted epitopes.

Item	Protein	Epitope	Types	Status	Status2	Lenth
1	CD2V-1	LATCGKAGNFCECSNYSTS	B-Cell	N	ASFV1	19
2	CD2V-2	YSRYQYNTPIYYMRP	B-Cell/T-cell	N	ASFV1	15
3	CD2V-3	DSNITNDNNDINGVSWN	T-cell	N	ASFV1	17
4	CD2V-4	LTPATPPNITYNCTNFLITCKKNNGTNT	T-cell	N	ASFV1	28
5	CD2V-5	ISIITFLSLRKRKKHVEEI	T-cell	N	ASFV1	19
6	CD2V-6	PLNPFPLPKPCPPPKPCPPPKPCPPPK	T-cell	N	ASFV1	27
7	EP153R-1	YNNVCYYFGNEEKNYNNASNYCKQLNS	T-cell	N	ASFV1	27
8	EP153R-2	NYWVNYSLIKNE	T-cell	N	ASFV1	12
9	P30-1	MEVIFKTDLRSSSQVVFHAG	B-Cell	N	ASFV1	20
10	P30-2	NMILHVLFEE	B-Cell	N	ASFV1	10
11	P30-3	ESSASSENIH	B-Cell	N	ASFV1	10
12	P30-4	TSSFETLFEQ	B-Cell	N	ASFV1	10
13	P30-5	QHIEQYGKAPDFNKV	B-Cell	N	ASFV1	15
14	P30-6	LKEEEKEVVRLMVIKLLKKNKL	B-Cell	N	N/A	22
15	P49-1	AGRGIPLGNPHVKPNIEQELIKS	B-Cell	N	ASFV1	23
16	P49-2	FPKDFNASSVPLTSAEKDHSLRGDNS	B-Cell	N	N/A	27
17	P49-3	GQAEYFDTSKQTISRHNNYIPKYTGGIGDS	B-Cell	N	N/A	30
18	P49-4	LADYRSDPPLWESDLPRHNRYSDNILN	B-Cell	N	ASFV1	27
19	P49-5	LNPQHKNIGYGDAQDLEPYS	B-Cell	N	ASFV1	20
20	P54-1	FFQPVYPRHYGECLSP	B-Cell	N	ASFV1	16
21	P54-2	SRKKKAAAIEEEDIQFINPYQDQQWV	B-Cell	N	ASFV1	26
22	P54-3	ATTASVGKPVTGRPATNRPATNKPVT	B-Cell	N	ASFV1	26
23	P72-1	GFEYNKVRPHTGTPTLGNKLT	B-Cell	N	ASFV1	21
24	P72-2	QMGAHGQLQTFPRNGYDWDNQTPLE	B-Cell	N	ASFV1	25
25	P72-3	YCEYPGERLYENVRFDVNGNSLDEYSSDVTTL	B-Cell	N	ASFV1	32
26	P72-4	HKPHQSKPILTDENDTQRTC	B-Cell	N	ASFV1	20
27	P72-5	VTHTNNNHHDEKLMS	B-Cell	N	ASFV1	15
28	P72-6	TWNISDQNPHQHRDWHK	B-Cell	N	ASFV1	17
29	P72-7	SFQDRDTALPDACSSISDI	B-Cell	N	ASFV1	19
30	P72-8	AINFLLLQNGSAVLRYS	T-cell	N	ASFV1	17
31	B602L-1	PLDRRIEAQRLDRKHI	B-Cell	N	ASFV1	16
32	B602L-2	VQKKYGGGEDCECTRV	B-Cell	N	ASFV1	16
33	B602L-3	INELKKEHTDKIQIVSKL	B-Cell	N	ASFV1	18
34	B602L-4	PKGQTRTLGSNRERERI	B-Cell	N	ASFV1	17
35	B602L-5	MSRIFRGDNALNMGRPKFLSDQIFNKV	B-Cell	N	ASFV1	27
36	B602L-6	NKALQKVGL	T-cell	N	ASFV1	9
37	B602L-7	HNKQEFQSY	T-cell	N	ASFV1	9
38	B602L-8	ITKTFVNNI	T-cell	N	ASFV1	9
39	B602L-9	DNAPAGHYY	T-cell	N	ASFV1	9
40	B602L-10	TPEEAAQRVY	T-cell	N	ASFV1	10

41	B602L-11	VNDALSTRW	T-cell	N	ASFV1	9
42	B602L-12	EFYQKLFSF	T-cell	N	ASFV1	9
43	B602L-13	IPIYLKENY	T-cell	N	ASFV1	9
44	B602L-14	YINQALHEL	T-cell	N	ASFV1	9

788

Supplementary file S2. Amino acid sequences of the antigens obtained from Pig/HLJ/2018 strain used to design mRNA vaccine:

mRNA-B602L

MDAMKRGLCCVLLLCGAVFVSPMAEFNIDELLKNVLEDPSTEISEETLKQLYQRTNPYKQFKNDSRVAFCSFTNLREQYIRRLIMTSFIGYVFKALQEWMPSYSKPTHTTKTLLSELITLVDTLKQETNDVPSESVVNTILSIADSCKTQTQKSKEAKTTIDSFLREHFVFDPNLHAQSAYTCADTNVDTCASMCADTNVDTCASMCADTNVDTCASTCTSTEYTDLADPERIPLHIMQKTLNVPNELQADIDAITQTPQGYRAAAHILQNIELHQSIKHMLENPRAFKPILFNTKITRYLSQHIPPQDTFYKWNYYIEDNYEELRAATESIYPEKPDLEFAFIIYDVVDSSNQQKVDEFYYKYKDQIFSEVSSIQLGNWTLLGSFKANRERYNYFNQNNEIIKRILDRHEEDLKIGKEILRNTIYHKKAKNIQETGPDAPGLSIYNSTFHTDSGIKGLLSFKELKNLEKASGNIKKAREYDFIDDCEEKIKQLLSKENLTPDEESELIKTKKQLDNALEMLNVPDDTIRVDMWVNNNNKLEKEILYTKAELHHHHHH

mRNA-CD2V

MIILIFLIFSTKPRNIVLSIDYWVSFNKTIILDSNITNDNNDINGVSWNFFNNSFNTLATCGKAGNFCECSNYSTSIYNITNNCSLTIFPHNDVFDTTYQVVWNQIINYTIKLLTPATPPNITYNCTNFLITCKKNNGTNTNIYLNINDTFVKYTNESILEYNWNNSNINNFTATCIINNTISTSNETTLINCTYLTLSSNYFYTFFKLYYIPLSIIIGITISILLISIITFLSLRKRKKHVEEIESPPPESNEEEQCQHDDTTSIHEPSPREPLLPKPYSRYQYNTPIYYMRPSTQPLNPFPLPKPCPPPKPCPPPKPCPPPKPCPSAESYSPPKPLPSIPLLPNIPPLSTQNISLIHVDRIITYQRTRALVHHHHHH

mRNA-EP153R

MRAWIFFLLCLAGRALATKPRMFSNKKYIGLINKKEGLKKKIDDYSILIIGILIGTNILSLIINIIGEINKPICYQNDDKIFYCPKDWVGYNNVCYYFGNEEKNYNNASNYCKQLNSTLTNNNTILVNLTKTLNLTKTYNHESNYWVNYSLIKNESVLLRDSGYYKKQKHVSLLYICSKTYQRTRALVHHHHHH

mRNA-P30

MRAWIFFLLCLAGRALATKPRMDFILNISMKMEVIFKTDLRSSSQVVFHAGSLYNWFSVEIINSGRIVTTAIKTLLSTVKYDIVKSARIYAGQGYTEHQAQEEWNMILHVLFEEETESSASSENIHEKNDNETNECTSSFETLFEQEPSSEVPKDSKLYMLAQKTVQHIEQYGKAPDFNKVIRAHNFIQTIYGTPLKEEEKEVVRLMVIKLLKKKTYQRTRALVHHHHHH

mRNA-P54

MRAWIFFLLCLAGRALATKPRMDSEFFQPVYPRHYGECLSPVTTPSFFSTHMYTILIAIVVLVIIIIVLIYLFSSRKKKAAAIEEEDIQFINPYQDQQWVEVTPQPGTSKPAGATTASVGKPVTGRPATNRPATNKPVTDNPVTDRLVMATGGPAAAPAAASAPAHPAEPYTTVTTQNTASQTMSAIENLRQRNTYTHKDLENSLTYQRTRALVHHHHHH

mRNA-P72

MRAWIFFLLCLAGRALATKPRMASGGAFCLIANDGKADKIILAQDLLNSRISNIKNVNKSYGKPDPEPTLSQIEETHLVHFNAHFKPYVPVGFEYNKVRPHTGTPTLGNKLTFGIPQYGDFFHDMVGHHILGACHSSWQDAPIQGTSQMGAHGQLQTFPRNGYDWDNQTPLEGAVYTLVDPFGRPIVPGTKNAYRNLVYYCEYPG

ERLYENVRFDVNGNSLDEYSSDVTTLVRKFCIPGDKMTGYKHLVGQEVSVEGTSGPLLCNIHDLHKPHQSKPILTDENDTQRTCSHTNPKFLSQHFPENSHNIQTAGKQDITPITDATYLDIRRNVHYSCNGPQTPKYYQPPLALWIKLRFWFNENVNLAIPSVSIPFGERFITIKLASQKDLVNEFPGLFVRQSRFIAGRPSRRNIRFKPWFIPGVINEISLTNNELYINNLFVTPEIHNLFVKRVRFSLIRVHKTQVTHTNNNHHDEKLMSALKWPIEYMFIGLKPTWNISDQNPHQHRDWHKFGHVVNAIMQPTHHAEISFQDRDTALPDACSSISDISPVTYPITLPIIKNISVTAHGINLIDKFPSKFCSSYIPFHYGGNAIKTPDDPGAMMITFALKPREEYQPSGHINVSRAREFYISWDTDYVGSITTADLVVSASAINFLLLQNGSAVLRYSTTYQRTRALVGGSGYIPEAPRDGQAYVRKDGEWVLLSTFLHHHHHH

Supplementary file S3. The gating strategy for flow cytometry of CD4 and CD8 T cells.

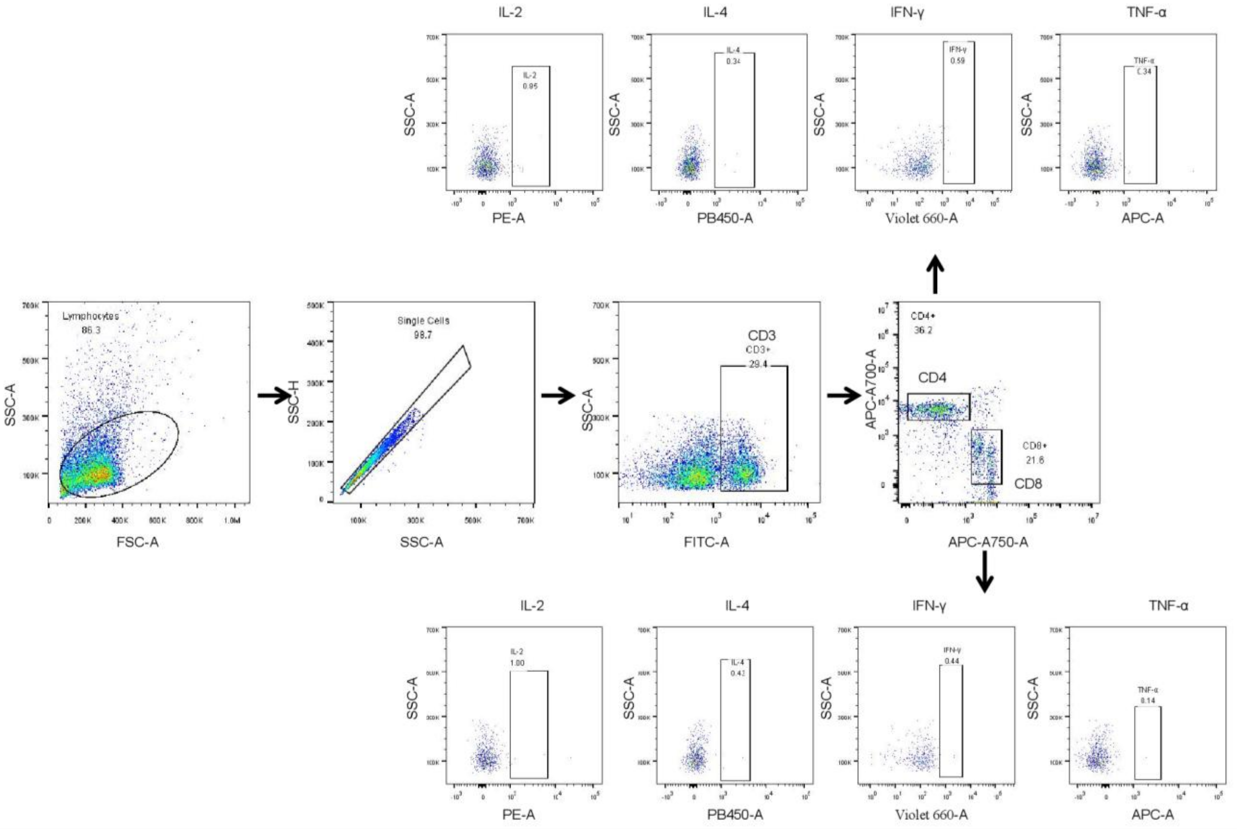

Supplementary file S4. The antibody clone information for each used antibody in flow cytometry.

Antibodies	Source	Clone
FITC anti-mouse CD3	BioLegend	17A2
Alexa Fluor® 700 anti-mouse CD4	BioLegend	GK1.5
APC/Fire™ 750 anti-mouse CD8a	BioLegend	53-6.7
APC anti-mouse TNF-α	BioLegend	MP6-XT22
Brilliant Violet 650™ anti-mouse IFN-γ	BioLegend	XMG1.2
PE anti-mouse IL-2	BioLegend	JES6-5H4
Brilliant Violet 421™ anti-mouse IL-4	BioLegend	11B11

Supplementary file S5. T cell responses to each individual antigen in mice.

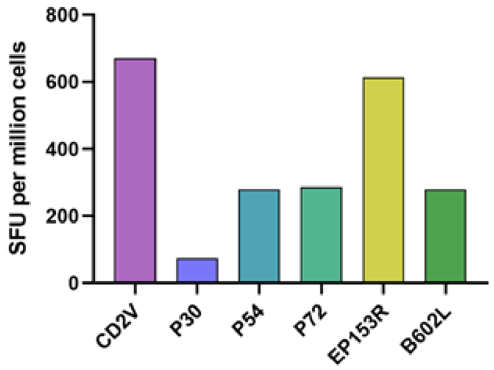

Supplementary file S6. Spot counts of IFN-γ-producing T cells detected by Elispot. IFN-γ secretion induced by stimulation of cells with the predicted T cell epitope peptides from each antigen was also assessed via ELISpot assay.

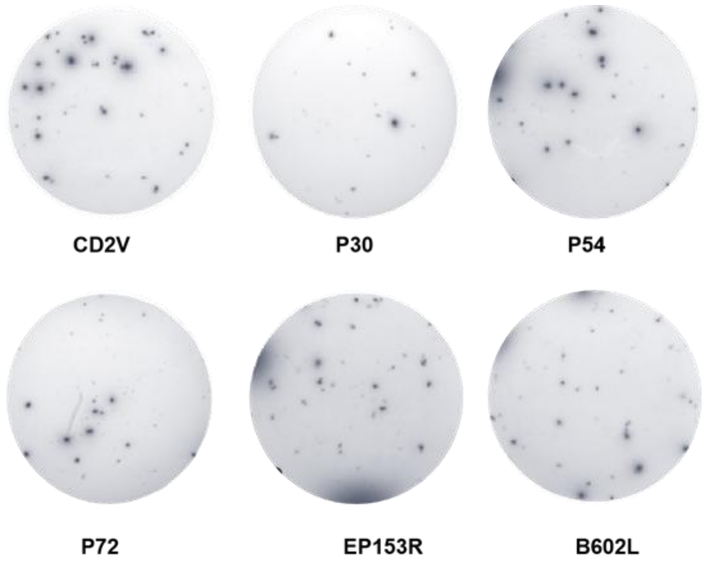

Supplement: Supplemental material — Two supplemental tables; supplemental data; three supplemental figures. [file spectrum.02909-24-s0001.pdf]
